# Arsenic and Heavy Metals in Vietnamese Rice: Assessment of Human Exposure to These Elements through Rice Consumption

**DOI:** 10.1155/2021/6661955

**Published:** 2021-01-22

**Authors:** Dinh Binh Chu, Hung Tuan Duong, Minh Thi Nguyet Luu, Hong-An Vu-Thi, Bich-Thuy Ly, Vu Duc Loi

**Affiliations:** ^1^Department of Analytical Chemistry, School of Chemical Engineering, Hanoi University of Science and Technology, 1 Dai Co Viet Hai Ba Trung, Hanoi 100000, Vietnam; ^2^Institute of Chemistry, Vietnam Academy of Science and Technology (VAST), 18 Hoang Quoc Viet Cau Giay, Hanoi 100000, Vietnam; ^3^School of Environmental Science and Technology, Hanoi University of Science and Technology, 1 Dai Co Viet Hai Ba Trung, Hanoi 100000, Vietnam

## Abstract

In this work, twelve heavy metals and arsenic, As, Cd, Co, Cr, Cu, Fe, Hg, Mn, Ni, Pb, Se, and Zn, in a rice sample collected from some areas of Vietnam have been quantified and implemented by using multiple analytical platforms such as ICP-MS, AAS, and mercury analyser. Seventy rice samples collected from the Red River Delta and mining zone activity were analysed. Concentration of heavy metals and arsenic in rice was analysed after appropriated sample digestion using internal or external calibration curves. The mean concentration (mg kg^−1^ dried weight) of the analysed elements in rice samples decreased on the order of Mn (19.268) > Fe (13.624) > Zn (8.163) > Cu (3.138) > Ni (0.384) > Cr (0.296) > Co (0.279) > As (0.115) > Cd (0.111) > Pb (0.075) > Hg (0.007) > Se (<LOD). Mercury, a highly toxic element, has been only found in rice samples collected in the mining activity zone (frequency detection 14.5% of total samples). The experimental results indicated that the heavy metals and arsenic found in rice collected from mining activity zone were higher than those in rice harvested from normal cultivated areas like the Red River Delta. The heavy metals and arsenic content in Vietnamese rice samples were also compared with the concentration of heavy metals in other foreign rice samples in some recent publications. The estimated daily intake through rice consumption was calculated and compared with the level proposed by the Food and Agriculture Organization of the United Nations. The results indicated that the provisional daily intake of Cd was higher than the level proposed by FAO, while the intake of other heavy metals was in an acceptable range of CODEX standard.

## 1. Introduction

In Vietnam, rice is the most important and widely cultivated crop, which is the main food product for internal consumption and also for export. Vietnam is among the top ten countries in the world that grow and export rice [1]. In addition, most of Vietnamese people are using cooked rice or rice-related products as meal every day as a traditional culture. As traditional culture, rice is mostly planted in the wetland. Therefore, rice can contain some heavy metals and toxic elements such as cadmium, lead, copper, selenium, mercury, arsenic, etc. by uptaking these elements from contaminated soil, agricultural irrigation water, pesticides, or other contaminated sources. Mechanism uptake of heavy metals and arsenic in rice and distribution of heavy metals in different parts of rice have been investigated [2], especially in nearby mining activity zones [3]. Heavy metals as one of the major contaminants in environment attract great attention due to their toxicology and nonbiodegradation and persistent natures. In addition, heavy metals can be accumulated along food chain and affect human health. To the best of our knowledge, only a few papers regarding analysis of these elements in Vietnamese rice samples have been published [4, 5]. In addition, the human exposure to these elements should be paid attention to, especially for habitants who live nearby mining activity zone. Therefore, it is necessary to have analytical methods with high selectivity and sensitivity for analysis of heavy metals and toxic elements in rice samples, especially in rice samples grown in mining activity area. In addition, obtaining information about concentration of heavy metals in food and its consumption as rice is very important in assessment of exposure to these elements in human health. Some recent publications have been indicated that ingestion on food contaminated with heavy metals like vegetable, rice was one of main sources of the human exposure to heavy metals [6, 7].

To date, many analytical methods like flame atomic absorption spectrometry (F-AAS) [8], graphite furnace atomic absorption spectrometry (GF-AAS) [9, 10], hydride generation atomic absorption spectrometry (HG-AAS) [11–13], and inductively coupled plasma optical emission spectrometry (ICP-OES) [14, 15] have been developed for analysis of heavy metals in the food matrices, especially in rice samples. Some speciation analysis methods have been also developed for analysis of toxic compounds, especially arsenic compounds, in rice samples [16–20]. However, inductively coupled plasma-dynamic reaction cell-quadrupole mass spectrometry (ICP-DRC-QMS) has become a golden standard for multielement analysis because of sensitivity, selectivity, wide dynamic linearity range, and isotopic analysis ability [21].

In this work, the multielemental analysis of heavy metals and some toxic elements in rice samples was implemented by using different analytical techniques such as flame atomic absorption spectrometry (F-AAS), inductively coupled plasma-dynamic reaction cell-quadrupole mass spectrometry (ICP-DRC-QMS), and cold vapour-atomic absorption spectrometry (CV-AAS based mercury analyser). Seventy rice samples were taken from different area production (the Red River Delta and mining activity zone) and concentration of arsenic and heavy metals was analysed after microwave-assisted acidic digestion. The validation of the developed methods was performed by rice-based matrix certified reference (ERM-BC 211 from European Commission and DORM 2 from NRC, Canada) materials and spiking experiments as well. The developed method was applied to the assessment of heavy metal contaminant in rice samples collected from some rice fields in Red River Delta and in mining activity zone in Vietnam. Besides, heavy metals and arsenic can be harmful to human health when foodstuff containing these elements is regularly used in the diet; therefore, human exposure to these elements through rice consumption was assessed and implemented.

## 2. Materials and Methods

### 2.1. Materials

Single element standard including arsenic (As, 1000 mg L^−1^), cadmium (Cd; 1000 mg L^−1^), cobalt (Co, 1000 mg L^−1^), chromium (Cr, 1000 mg L^−1^), copper (Cu, 1000 mg L^−1^), iron (Fe, 1000 mg L^−1^), manganese (Mn, 1000 mg L^−1^), mercury (Hg, 1000 mg L^−1^), nickel (Ni 1000 mg L^−1^), lead (Pb, 1000 mg L^−1^), selenium (Se, 1000 mg L^−1^), zinc (Zn, 1000 mg L^−1^), and indium (In, 1000 mg L^−1^) for ICP-MS were purchased from Sigma Aldrich (Singapore). HNO_3_, HCl, H_2_SO_4_, and HClO_4_ were collected from Merck (Singapore, Suprapur grade). Other chemicals were purchased from Sigma Aldrich (Singapore, analytical grade). Deionised water was purified by Milli-Q purify system (Millipore, Singapore). The working standard solution was prepared by dilution of stock solution in diluted nitric acid. Indium standard solution for ICP-MS from Sigma Aldrich (Singapore) was used as an internal standard for ICP-DRC-QMS measurement. Rice-based certified reference material, ERM-BC 211 certified for arsenic, and fish-based certified reference material, DORM 2 certified for mercury, were purchased from Institute for Reference Materials and Measurements and European Commission and National Research Council, Canada, respectively.

### 2.2. Analytical Methods and Sample Preparation

#### 2.2.1. ICP-DRC-MS Measurement

A NexION 2000 ICP-DRC-QMS (Perkin Elmer, Ottawa, Canada) was used for multielemental analysis. The sensitivity and selectivity of the ICP-DRC-QMS were daily checked by using a specific tuning solution from Perkin Elmer (Perkin Elmer, USA). Sensitivity of all target elements was optimized and daily checked by using in-house mixture standard solution (10 ng mL^−1^ for all target analytes). The optimum operating conditions of ICP-DRC-MS are shown in Table S1 in the Supplementary Information. All target analytes were measured in kinetic energy discrimination (KED) and dynamic reaction cell (DRC) mode employing with helium as collision gas in order to eliminate polyatomic ion interferences exception of cadmium, arsenic, and selenium. For determination of arsenic and selenium, the dynamic reaction cell mode ultilizing with oxygen as a reaction gas was used to overcome polyatomic ion ^40^Ar^35^Cl^+^ on arsenic measurement and argon-based polyatomic ion interferences in selenium measurement. The standard mode was employed for cadmium measurement.

#### 2.2.2. AAS Measurements

Because of high concentration and polyatomic mass interferences in ICP-MS measurement, concentration of Mn and Fe in rice samples was measured by flame atomic absorption spectrometry using acetylene and compressed air as fuel gas and oxidation gas, respectively. The optimum operating conditions for analysis of Fe and Mn by F-AAS are listed in Table S2 in the Supplementary Information.

#### 2.2.3. Mercury Analysis

Total mercury in rice samples was determined by cold vapor atomic absorption spectrometry (CV-AAS) according to the method of Hirokatsu Akagi with some modifications [22]. In brief, the method involves digestion with HNO_3_, HClO_4_, and H_2_SO_4_ followed by reduction to Hg^0^ by SnCl_2_ and quantification by cold vapor atomic absorption spectrometry (CVAAS). Automatic Mercury Analyzer Hg-201 (Sanso Company) was used. After digestion, 5 mL of sample was added in the reaction vial with the addition of 1 mL of stannous chloride (SnCl_2_-10%) to reduce Hg^2+^ to Hg^0^. Then, the Hg vapor generated is conducted into a flask containing NaOH at 5 N, which neutralizes the acid vapours, and a flask containing an ice bath, in which water vapour is condensed. Thereupon, the Hg vapours are transported to the photon-absorption cell to measure absorbance of 253.7 nm. In the analysis of total Hg, concentration of total Hg in sample solution was calculated by calibration curve.

#### 2.2.4. Rice Sampling Sites

**Figure 1:**
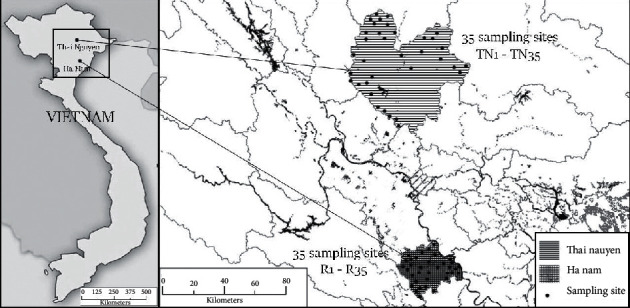
Maps of sampling sites, Thai Nguyen and Ha Nam provinces.

Rice samples were collected from different sites including the rice fields in the Red River Delta and activity mining zone in 2018 and 2019 harvest seasons. Rice samples were coded TN1-TN35 and R1-R35 collected from mining activity areas (Thai Nguyen province) and Red River Delta (Ha Nam province), respectively. The maps of both sampling sites are shown in Figure 1. The rice samples were also collected from both dry and wet seasons. Rice samples were rinsed using deionized water and air drying. Rice samples then were kept in plastic zip-bag and stored at 4°C until chemical analysis.

#### 2.2.5. Sample Preparation

*(1) For ICP-DRC-QMS and F-AAS Measurement*. Dried rice sample was grilled in the food processor and re-grilled on the agate mortar and pestle. 0.2000 gram of rice was weighted on the analytical balance and transferred into microware PPFE vessel and subjected for sample digestion by microwave oven. In brief, approximately 2 mL of concentrated nitric acid (65%) and 1.0 mL of hydrogen peroxide (34% by volume) were poured into digestion vessel. Afterward, the rice sample was digested in dedicated microwave oven (Multiwave PRO, Anton PAAR, Graz, Austria) by controlled temperature program. After complete digestion, the vessels were cooled to 50°C. Samples were then cooled to room temperature and transferred to 50 mL volumetric flasks and made up to volume with deionised water. These solutions were then analysed by ICP-DRC-QMS using In as internal standard (concentration of internal standard in final solution was 10 ng mL^−1^).

*(2) For CV-AAS Measurement of Mercury.* Technically, approximately 100 mg of each rice sample was weighed in a 50 mL digestion tube, and 1 mL of deionized water was added plus 2 mL mixture of concentrated HNO_3_ and HClO_4_ (1 /1: v/v) and 5 mL of H_2_SO_4_ and heated for 30 minutes in a hot plate at temperature of 230°C. After cooling, the solution was transferred into 50 mL volumetric flask and made up with deionized water to a final volume of 50 mL. The final digestion solution was subjected for analysis of mercury by CV-AAS.

#### 2.2.6. Quality Control

For the quality control, rice-based certificated reference material (ERM-BC 211 from European Reference Materials, Belgium) was used for analysis of arsenic. DORM-2 was used for validation of analytical method for mercury and spiking experiments for other elements. Digestion of the CRM was prepared by the same preparation procedure for the rice samples. The total number of blank and quality control samples contained at least 20% of total samples analysis for each analysis batch.

### 2.3. Data Analysis

The data analysis was performed by using data analysis function in the Microsoft Excel version 2020 (Microsoft, USA). The confident interval level was set at 95%. The human exposure to these elements was calculated as suggestion of FAO.

The average estimated daily intake of these compounds for the Vietnamese consumer through the consumption of rice and rice products was calculated according to equation (1) as proposal from Food and Agriculture Organization of the United Nations (FAO) [23].

(1) $$\begin{array}{c} {\text{EDI}}_{\text{rice}}=\;\frac{\text{rice consumption }\left(\text{kg day}-1\right)*\text{concentration }\left(\text{mg kg}-1\right)}{\text{body weight }\left(\text{kg}\right)}. \end{array}$$

EDI_rice_ is the estimated daily intake of these elements (mg day^−1^ kg^−1^ body weight) through rice consumption. Mean values of body weight for Vietnamese adult man and woman are 58 kg and 45 kg, respectively. The average rice consumption of Vietnamese people is 589 gram per person per day [24]. The average estimated daily intake of heavy metals was calculated based on the average and range concentration of these elements in rice sample and rice consumption.

## 3. Results and Discussion

### 3.1. Analytical Figure of Merits of Multianalytical Techniques for Analysis of Rice Samples

Seven independent solutions (from 1.000 to 50.000 *μ*g/kg) including multielements such as Cd, Zn, Cu, Pb, Ni, Cr, Mo, Se, As, and In as internal standard (final concentration of In in solution 10 *μ*g kg^−1^) were prepared in diluted nitric acid. All elements were analysed by ICP-MS as indicated in Table 1. Indium was used as an internal standard and the intensity ratio was calculated and fitted as a function of concentration. Because of using In as internal standard, the robustness of the ICP-MS measurement was improved in terms of repeatability and reproducibility [25]. In case of Fe and Mn, the atomic absorption spectrometry was used because of high concentration in rice samples and polyatomic ion interferences in ICP-MS (^40^Ar^16^O^+^ on ^56^Fe^+^ measurement and ^40^Ar^14^N^1^H^+^, ^39^K^16^O^+^, ^40^Ar^15^N^+^, etc. on ^55^Mn^+^ measurement) [26]. In addition, Hg was analysed by cold vapour-atomic absorption spectrometry in order to achieve high selectivity and sensitivity. The linearity range, correlation coefficient, LOD, and LOQ of the analytical methods are listed in the Table 1.

As can be seen in Table 1, the sensitivity of the introduced analytical methods was high enough for direct quantification of the target elements in rice samples after microwave-assisted acidic digestion. In addition, validation of analytical methods for analysis of rice samples was performed via spiking experiments. The recovery of these elements was in the range from (80 ± 7.3) % to (118 ± 15.3) %. In case of As and Hg, the recovery of the sample preparation was performed by using certified reference materials. ERM-BC211 and DORM 2 certified reference materials were used for validation of As and Hg, respectively. The recoveries of As and Hg were corresponding to (99 ± 5.5) % and (90 ± 7.8) %.

### 3.2. Application for Analysis of Real Samples

**Figure 2:**
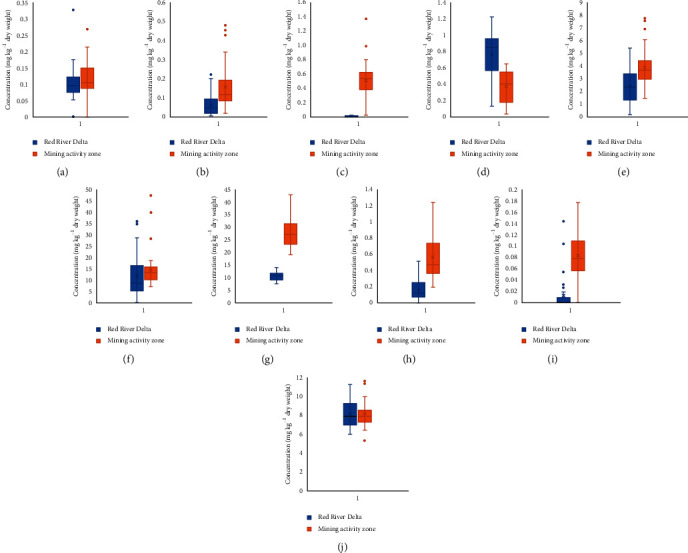
The whisker and box diagram of the heavy metals and arsenic in rice in both sampling areas. (a) As. (b) Cd. (c) Co. (d) Cr. (e) Cu. (f) Fe. (g) Mn. (h) Ni. (i) Pb. (j) Zn.

Rice samples were analysed after acidic microwave-assisted digestion using oven or other preparation methods as mentioned before. In total, 70 rice samples were analysed and the concentration of these elements in rice samples is reported in Table S3 in the Supplementary Information. The concentration unit of all elements in Table S3 was presented in milligram of heavy metal per kg dried weight of sample (mg kg^−1^). The comparison of the concentration of each element in both sampling areas is shown in Figure 2. The average value and range of the concentration of the analysed elements in both subrice samples, collected in Red River Delta and in mining activity zone, are also calculated and presented in Table 2. In addition, the analysed data in this study are also compared with some recent publications as indicated in this table.

The total content of heavy metals and arsenic in the analysed rice samples is presented in Table S3 in the Supplementary Information. Concentration of Mn in rice gain was the highest followed by the concentration of Fe. Thus, the mean concentration (mg kg^−1,^ dried weight) of the elements in rice samples decreased on the order of Mn (19.268) > Fe (13.624) > Zn (8.163) > Cu (3.138) > Ni (0.384) > Cr (0.296) > Co (0.279) > As (0.115) > Cd (0.111) > Pb (0.075) > Hg (0.007) > Se (<LOD).

As can be clearly seen in Table S3, the mean values of concentration of heavy metals and arsenic in two sub-rice samples collected from Red River Delta and mining activity zone were significantly different. It was recently reported that the elevated concentration of Pb and other elements was found in the mining activity zone in Vietnam [32]. Concentration of most analysed heavy metals in rice samples from mining activity zone was higher than that in rice collected from Red River Delta. For instance, the average concentration of Cd in rice samples collected from mining activity zone was approximately 2.5 times higher than that in rice samples taken from Red River Delta. However, the mean concentration of Zn was similar in two sub-samples. Concentration of Se in all collected rice samples was below detection limit. In case of mercury, few samples collected from mining activity zone were detected at a very low concentration. The average concentration of As and heavy metals rice samples associated with range values is shown in Table 2. Concentration of elements in Vietnamese rice samples is similar to those of the foreign rice samples as shown in this table. In terms of comparison with concentration of heavy metals in Vietnam rice samples, in this study, it was higher than that reported in previous publications. It could be explained that rice samples in previous publication were only taken in the Red River Delta [5] or a small number of rice samples were analysed (only 3 rice samples) [31].

### 3.3. Assessment of Daily Intake of These Elements through Rice Consumptions

The estimated daily intake of these elements through rice consumption was calculated by using equation (1) and presented in the Table 3.

#### 3.3.1. Arsenic

As, as a metalloid, is toxic to plants and animals as well. Rice absorbs As more efficiently than other cereals, mainly from contaminated paddy soil and irrigation water and other sources like pesticides or fertilizers. Rice grown on the As contaminated paddy soil can accumulate elevated levels of arsenic and increase the transfer of it from soil to rice. In addition, maximum residual level of inorganic arsenic in rice is below 0.2 mg kg^−1^ according to European Council and USA Food and Drug Administration regulars and Vietnam standard has been set a maximum concentration of total As in rice as well (total arsenic below 1.0 mg kg^−1^). As can be seen in Table S3 in Supplementary Materials, the average concentration of As in 70 rice samples is 0.115 mg kg^−1^ and range is from 0.052 mg kg^−1^ to 0.328 mg kg^−1^. All analysed rice samples containing arsenic were below Vietnam national standard. However, only a few rice samples (4 samples, approximately 5.7% of the total collected samples) containing As were of higher maximum level according to European Council and USA Food and Drug Administration. Moreover, the speciation analysis of As-related compounds in these rice samples should be taken into account in order to distinguish inorganic and organic arsenic species. The mechanism uptake of total arsenic and arsenic compounds in rice species was summarized in a recent publication [33]. Several factors such as cropping seasons, rice species, and type of soils (phosphorus, sulphur, iron, manganese, etc. content) affect the uptake of arsenic in rice.

Humane exposure to arsenic may include inhalation of dust in the air, water and food consumption, and dermal contact of soil and water. The estimated daily intake of As from rice consumption grown in the mining activity zone and in Red River Delta is shown in Table 3. The estimated daily intake of As for man and woman was 0.0012 mg day^−1^ kg^−1^ (range 0.0005–0.0033 mg day^−1^ kg^−1^) and 0.0015 mg day^−1^ kg^−1^ (range 0.0007–0.0043 mg day^−1^ kg^−1^). The provisional tolerable weekly intake (PTWI) of arsenic according to CODEX standard is 0.0021 mg kg^−1^ body weight per day. Therefore, estimated daily intake of arsenic through rice consumption in this study was lower than CODEX standard.

#### 3.3.2. Cadmium

In this study, the concentration of Cd in 70 rice samples ranged from 0.005 to 0.480 mg kg^−1^ and mean concentration was 0.111 mg kg^−1^. The average concentration of Cd in 70 rice samples was below CODEX standard for polished rice (0.4 mg kg^−1^ dried weight) with the exception of three samples collected from mining activity zone (TN8, TN11, and TN12) [34, 35]. In addition, the average concentration of cadmium in 35 rice samples collected from mining activity zone is approximately 2.5 times higher than the concentration in rice collected in the Red River Delta as aforementioned. The mechanism uptake of Cd in rice planted on contaminated soil has been summarized in the study of Hui Li et al. [33]. The average estimated daily intake of Cd through rice consumption for man and women was 0.0011 mg day^−1^kg^−1^ and 0.0014 mg day^−1^kg^−1^, respectively. The potential of human exposure to Cd through rice consumption harvested in mining activity zone was also higher than other areas. The provisional tolerable daily intake of Cd is 0.00082 mg kg^−1^ according to CODEX. Therefore, the estimated daily intake of Cd through rice in this study was higher than that from CODEX standard. Therefore, the action for mitigation of such element uptake in rice like immobilization in soil, bioremediation, and molecular biotechnology should be paid attention to.

#### 3.3.3. Chromium

Cr was detected in 74.5% of rice samples (a total of 70) with mean concentration 0.296 mg kg^−1^ and ranged from 0.004 to 1.223 mg kg^−1^. Other previous studies showed that the linearity correlation between concentration of chromium in rice and spiked soil has been observed [36, 37]. That means the main contribution of chromium in rice can come from agricultural contaminated soil. Due to high toxicology of Cr, especially of hexavalent chromium compounds, the provisional tolerance daily intake of total chromium was defined as 0.0033 mg kg^−1^day^−1^ [38]. The estimated daily intake of Cr through rice consumption was 0.003 mg d^−1^ kg^−1^ (0.0001–0.0124 mg day^−1^ kg^−1^) and 0.0039 mg day^−1^ kg^−1^ (0.0001–0.0160 mg day^−1^ kg^−1^) for Vietnamese woman and man, respectively.

#### 3.3.4. Cobalt

The average concentration of Co in 70 rice samples was 0.275 mg kg^−1^ and ranged from 0.001 mg kg^−1^ to 1.369 mg kg^−1^. The average concentration of Co in the Red River Delta rice samples was 0.008 mg kg^−1^ (0.001–0.018 mg kg^−1^). Meanwhile, the average concentration of such element in rice sampled in mining activity zone was 0.512 mg kg^−1^ (0.021–1.369 mg kg^−1^). As a result, the concentration of Co in rice samples collected from mining activity zone was higher than that in the Red River Delta rice samples. It could be explained by accumulation of such element in rice from irrigation water, contaminated soil, or other sources in mining activity area [39]. The concentration of Co in these samples was also higher than that reported in a previous study [31]. The average estimated daily intakes of Co for Vietnamese woman and man were 0.0037 mg day^−1^ kg^−1^ and 0.0028 mg day^−1^ kg^−1^. Moreover, no literature reported the provisional tolerance daily intake of such element to human health.

#### 3.3.5. Copper

Cu was detected in all collected rice samples with average concentration 3.138 mg kg^−1^ and range 0.118 to 7.754 mg kg^−1^. The average concentration of Cu in this study was higher than that in Vietnamese rice samples reported from previous publication [5]. However, Cu content in these rice samples was the same concentration level as other rice samples collected in other countries as shown in Table 2. The estimated daily intake of Cu through rice consumption for Vietnamese woman and man was 0.0411 mg day^−1^ kg^−1^ and 0.0319 mg day^−1^ kg^−1^, respectively.

#### 3.3.6. Lead

The average concentration of Pb in all analysed rice samples was 0.075 mg kg^−1^ and range was from 0.03 to 0.177 mg kg^−1^. Concentration of Pb in all analysed rice samples was below maximum residual level according to CODEX standard (0.2 mg kg^−1^ dried weight for whole commodity). The concentration of Pb in rice samples collected from mining activity zone was also higher than that in the Red River Delta. The high concentration of Pb contained in rice collected in/nearby mining activity zone could be explained by transfer of this element from agricultural soil to rice in these areas [32, 40]. The average estimated daily intake (max-min) of man and women was 0.0008 mg day^−1^ kg^−1^ (0.0001–0.0018 mg day^−1^ kg^−1^) and 0.001 mg day^−1^ kg^−1^ (0.0001–0.0023 mg day^−1^ kg^−1^), respectively. The estimated daily intake of Pb through rice consumption was lower than intake level according to CODEX (0.003 mg day^−1^ kg^−1^).

#### 3.3.7. Manganese

The mean Mn concentration in rice samples was 19.19 mg kg^−1^ and the range was from 7.4 to 42.65 mg kg^−1^. The higher concentration of Mn (27.92 mg kg^−1^, mean value) was found in the rice samples collected from mining activity zone compared to concentration of this element in Red River Delta rice samples. In addition, Mn concentration in rice samples in this study was higher than that in previous publications but it was lower than that in Vietnamese brown rice [5].

#### 3.3.8. Nickel

In this study, the average concentration of Ni in 70 rice samples was 0.384 mg kg^−1^ (range from DL to 1.234 mg kg^−1^). Compared to previous study, Phuong et al. reported that the mean concentration of Ni in Vietnamese white rice samples was 0.869 mg kg^−1^. That means the finding of this study about concentration of Ni was three times lower. The concentration of Ni detected in rice in this study was lower than that in Chinese white rice in terms of comparison.

#### 3.3.9. Zinc

The average concentration of Zn in rice samples in this study was 8.155 mg kg^−1^. The range of Zn concentration was from 5.341 to 11.653 mg kg^−1^. Interestingly, the concentration of Zn in rice samples collected from both areas, in the Red River Delta and in mining activity area, was the same concentration level. Compared with Zn in Korean white rice, Thai white rice, and Australian white rice, the level of Zn in Vietnamese white rice was two times lower. However, the concentration of Zn in Vietnamese white rice and Chinese white rice was observed to be at the same level. The estimated daily intake of Zn through rice consumption for Vietnamese woman and man was 0.1068 mg day^−1^ kg^−1^ and 0.0826 mg day^−1^ kg^−1^, respectively.

#### 3.3.10. Other Elements

In this work, the concentration of Se and Hg was also determined in rice samples. In case of selenium, the concentration of selenium in all rice samples was below method detection. Meanwhile, mercury at low concentration was presented in a few rice samples, all of which collected from mining activity area. Therefore, determination of mercury in a large number of rice samples collected from mining activity zone as well as in contaminated soil, or irrigation water, should be conducted in order to have reliable evidence to confirm. The estimated daily intake of Se and Hg was not also presented in this study.

## 4. Conclusion

In this study, the concentration of heavy metals such as Cd, Co, Cr, Cu, Fe, Mn, Ni, Pb, and Se and toxic elements like arsenic and mercury in rice samples collected from Red River Delta has been analysed and presented. The finding in this study indicated that the concentration of all analysed heavy metals in rice samples grown in mining activity area was higher than the concentration of heavy metals in rice samples collected in the Red River Delta except Se and Zn. In addition, the human exposure to these elements has also been investigated and implemented. The provisional daily intake of all investigated heavy metals was in an acceptable range according to CODEX standard except Cd and As. Further investigation of human exposure to two elements, Cd and As, should be conducted regarding the chemical forms, mechanism uptake in the rice species, and toxicology of these elements.

## Figures and Tables

**Table 1:** Characteristics of the analytical method for the analysed rice samples.

No	Elements	Method	Linearity range (*μ*g/kg)	*R* ^2^	LOD (mg kg^−1^)	LOQ (mg kg^−1^)
1	As	ICP-DRC-QMS	1.000–50	0.9999	0.0005	0.0017
2	Cd	ICP-MS	1.000–50	0.9998	0.0002	0.0007
3	Co	ICP-MS	1.000–50	0.9997	0.0013	0.0042
4	Cr	ICP-MS	1.000–50	0.9999	0.0218	0.0726
5	Cu	ICP-MS	1.000–50	0.9999	0.0025	0.0084
6	Fe	AAS	50–5000	0.9994	0.016	0.052
7	Hg	CV-AAS	0.05–1.0	0.9994	0.0001	0.0003
8	Mn	AAS	100–5000	0.9988	0.013	0.04
9	Ni	ICP-MS	1.000–50	0.9999	0.0036	0.0121
10	Pb	ICP-MS	1.000–50	0.9998	0.0028	0.0095
11	Se	ICP-DRC-QMS	1.000–50	0.9999	0.00420	0.0140
12	Zn	ICP-MS	1.000–50	0.9995	0.0727	0.2424

**Table 2:** Comparison of the mean and range concentration of heavy metals in the analysed rice samples with data available from some previous studies.

Elements	Vietnamese white rice (*n* = 70)^*∗*^		Vietnamese white rice (*n* = 31) [5]		Korean white rice (*n* = 40) [27]		Chinese white rice [28–30]		Thai white rice (*n* = 12) [31]		Australian white rice (*n* = 21) [27, 31]	
	Mean	Min-max	Mean	Min-max	Mean	Min-max	Mean	Min-max	Mean	Min-max	Mean	Min-max
As	0.115	0.052–0.328	0.208	0.032–0.465	0.247	0.104–0.774	0.241	0.129–0.434	—	—	—	—
Cd	0.109	0.05–0.480	0.08	<0.003–0.048	0.174	0.010–0.980	0.283	0.013–2.066	0.013	0.006–0.018	0.008	0.007–0.017
Co	0.275	DL-1.369	0.007	0.006–0.008	—	—	—	—	—	—	0.021	0.007–0.042
Cr	0.304	DL-1.223	—	—	—	—	0.571	0.019–4.583	—	—	0.413	0.061–0.743
Cu	3.138	0.118–7.754	2.6	1.1–5.8	4.69	2.00–29.6	2.013	0.298–3.958	3.5	2.0–6.8	2.9	1.0–9.4
Fe	14.615	7.052–47.134	6.5	4.6–11.2	—	—	—	—	—	—	—	—
Hg	0.007	DL-0.011	—	—	—	—	—	—	—	—	—	—
Mn	19.19	7.444–42.650	9.9	5.9–16.3	—	—	17.314	2.629–33.049	7.9	6.2–10.0	24.4	9.2–51.7
Ni	0.384	LOD-1.243	0.869	0.1–2.022	—	—	1.332	0.015–7.352	—	—	0.166	0.0.061–0.356
Pb	0.075	0.03–0.177	0.002	—	0.804	0.010–3.34	0.145	0.009–1.959	0.419	0.096–0.921	0.375	0.002–1.248
Se	<DL	<DL	—	—	—	—	—	—	—	—	—	—
Zn	8.155	5.341–11.653	—	—	16.8	7.28–38.0	6.995	16.043–98.771	17.8	14.1–22.4	17.1	10.9–24.5

^*∗*^Data analysed in this study. DL: detection limit. *n*: number of rice samples, concentration unit: mg kg^−1^ dry weight.

**Table 3:** Estimated daily intake of arsenic and heavy metals through rice consumption.

No	Element	Estimated daily intake (mg day^−1^ kg^−1^ body weight)			
		Woman		Man	
		Mean value	Range	Mean value	Range
1	As	0.0015	0.0007–0.0043	0.0012	0.0005–0.0033
2	Cd	0.0014	0.0001–0.0063	0.0011	0.0001–0.0049
3	Co	0.0037	0.0001–0.0179	0.0028	0.0001–0.0139
4	Cr	0.0039	0.0001–0.016	0.003	0.0001–0.0126
5	Cu	0.0411	0.0015–0.1015	0.0319	0.0012–0.0787
6	Fe	0.1736	0.0026–0.6193	0.1347	0.002–0.4805
7	Hg	—	—	—	—
8	Mn	0.2522	0.0974–0.2522	0.1957	0.0756–0.4331
9	Ni	0.005	0.0001–0.0163	0.0039	0.0001–0.0126
10	Pb	0.001	0.0001–0.0023	0.0008	0.0001–0.0018
11	Se	—	—	—	—
12	Zn	0.1068	0.0699–0.1525	0.0826	0.00542–0.183

## Data Availability

The data used to support the findings of this study are included within the article.

## Supplementary Materials

Supplementary Materials Table S1: operating conditions of ICP-DRC-QMS for multielement analysis. Table S2: operating conditions of F-AAS for measurement of Fe and Mn. Table S3: concentration of analysed elements (mg kg^−1^ dry weight) in rice samples.
