# Supplementary material for: Arsenic and Heavy Metals in Vietnamese Rice: Assessment of Human Exposure to These Elements through Rice Consumption
Source: J Anal Methods Chem. 2021 Jan 22;2021:6661955. doi: 10.1155/2021/6661955 (PMC7846392; doi:10.1155/2021/6661955)
Supplement: Supplementary Materials — Table S1: operating conditions of ICP-DRC-QMS for multielement analysis. Table S2: operating conditions of F-AAS for measurement of Fe and Mn. Table S3: concentration of analysed elements (mg kg−1 dry weight) in rice samples. [file 6661955.f1.pdf]

Supplementary Information

Arsenic and heavy metals in Vietnamese rice: Assessment of human exposure to these elements through rice consumption

Dinh Binh Chu[1,*], Hung Tuan Duong[2], Minh Thi Nguyet Luu[2], Hong-An Vu-Thi[1], Bich-Thuy Ly[3], Vu Duc Loi[2]

1. Department of Analytical Chemistry, School of Chemical Engineering, Hanoi University of Science and Technology, 1 Dai Co Viet, Hai Ba Trung, Hanoi 100000, Vietnam
2. Institute of Chemistry, Vietnam Academy of Science and Technology (VAST), 18 Hoang Quoc Viet, Cau Giay, Hanoi 100000, Vietnam
3. School of Environmental Science and Technology, Hanoi University of Science and Technology, 1 Dai Co Viet, Hai Ba Trung, Hanoi 100000, Vietnam

Table S1. Operating condition of ICP-DRC-QMS for multielement analysis

**Parameters**	**Value**
ICP-MS	Nexion 2000 ICP-DRC-QMS (Perkin Elmer, Ottawa, Canada)
RF power	1600W
Plasma gas flow	15 L min^{-1}
Auxiliary gas flow	1.2 L min $^{-1}$
Nebulizer gas	1 L min^{-1}
Nebulizer	PFA
Spray chamber	Quartz cyclonic
Sample cone	Platinum sample cone
Skimmer cone	Platinum hyper skimmer cone
Cell entrance voltage	-7 V
Cell exit voltage	-7 V
DRC mode	Dynamic reaction cell with oxygen gas, 0.7 mL min^{-1}
Monitored ions	$^{75}As^{16}O^+$ (m/z 91); $^{111}Cd^+$; $^{59}Co^+$; $^{52}Cr^+$; $^{65}Cu^+$; $^{60}Ni^+$; $^{208}Pb^+$; $^{78}Se^{16}O^+$ (m/z 94); $^{66}Zn^+$ and $^{115}In^+$.
Measurement repeatability	3 times
Reading	5 times
Polyatomic interference correction	on
Measurement mode	Peak hopping
Dwell time	200 ms
RPq	0.6
Quantification mode	Internal calibration curve, $^{115}In^+$ as an internal standard

Table S2. Operating conditions of F-AAS for measurement of Fe and Mn

**Parameters**	**Value**
AAS	AA900 (Perkin Elmer, Ottawa, Canada)
C2H2 flow rate	2.2 L min^{-1} for Fe and 2.0 L min^{-1} for Mn
Compress air flow rate	10 L min^{-1}
HCL current for Fe	25 mA
HCL current for Mn	15 mA
Slit width	0.2 nm
Wave length for Fe	248.3 nm
Wave length for Mn	279.5 nm
Quantification mode	Peak height
Quantification	External calibration

Table S3 Concentration of analysed elements (mg kg^{-1} dry weight) in rice samples

No	Sample code	As	Cd	Co	Cr	Cu	Fe	Hg	Mn	Ni	Pb	Se	Zn
1	R1	0.081	0.096	0.001	0.067	2.312	6.3	< DL	10.975	0.164	0.144	< DL	6.641
2	R2	0.087	0.021	0.001	0.063	0.683	7.8	< DL	7.444	0.047	< DL	< DL	6.011
3	R3	0.073	0.013	< DL	< DL	1.013	3.6	< DL	8.699	0.067	< DL	< DL	7.076
4	R4	0.081	0.092	0.001	0.178	2.673	15.6	< DL	11.566	0.147	< DL	< DL	6.667
5	R5	0.055	0.201	< DL	< 0.001	3.385	6.4	< DL	12.332	0.510	< DL	< DL	7.913
6	R6	0.083	0.067	0.004	0.141	2.239	14.6	< DL	10.586	0.103	< DL	< DL	6.846
7	R7	0.061	0.035	0.001	< 0.001	1.712	8.2	< DL	8.762	0.153	< DL	< DL	6.938
8	R8	0.063	0.088	< DL	0.004	2.637	16.2	< DL	10.941	0.119	< DL	< DL	7.446
9	R9	0.052	0.222	0.001	0.004	3.038	35.7	< DL	11.590	0.277	< DL	< DL	11.004
10	R10	0.080	0.009	< DL	0.011	1.102	16.6	< DL	8.852	0.095	< DL	< DL	6.486
11	R11	0.328	0.114	0.017	0.999	3.801	14.6	< DL	12.846	0.420	< DL	< DL	10.217
12	R12	0.089	0.013	0.001	< 0.001	0.982	13.5	< DL	7.480	0.069	< DL	< DL	7.108
13	R13	0.063	0.055	0.003	0.293	2.107	18.1	< DL	11.735	0.192	0.010	< DL	9.458
14	R14	0.064	0.158	0.005	< 0.001	3.453	7.1	< DL	12.718	0.269	0.013	< DL	8.778
15	R15	0.069	0.153	0.003	0.013	2.315	22.7	< DL	10.585	0.326	0.008	< DL	9.443
16	R16	0.109	0.009	< DL	< 0.001	1.437	12.2	< DL	8.617	0.084	< DL	< DL	8.218
17	R17	0.087	0.055	0.009	0.598	1.463	5.5	< DL	9.934	0.364	< DL	< DL	8.568
18	R18	0.096	0.018	0.005	< 0.001	0.950	13.0	< DL	8.566	0.247	0.027	< DL	7.448
19	R19	0.122	0.008	0.011	< 0.001	2.098	8.7	< DL	9.151	< 0.001	< DL	< DL	9.536
20	R20	0.122	0.018	0.011	0.135	2.629	34.7	< DL	9.882	0.075	< DL	< DL	8.086
21	R21	0.095	0.021	0.008	< DL	2.867	11.9	< DL	11.128	0.093	< DL	< DL	9.318
22	R22	0.090	0.018	0.014	0.160	3.859	1.3	< DL	9.282	0.095	0.054	< DL	6.999
23	R23	0.132	0.047	0.002	0.059	5.405	5.3	< DL	11.682	< 0.001	< DL	< DL	8.895
24	R24	0.098	0.068	0.017	0.040	3.719	0.2	< DL	14.012	0.153	< 0.001	< DL	9.098
25	R25	0.151	0.027	0.006	0.043	3.310	22.8	< DL	9.301	0.144	0.032	< DL	8.758
26	R26	0.113	0.037	0.013	0.376	3.121	3.6	< DL	10.144	0.300	< DL	< DL	9.258
27	R27	0.105	0.111	0.017	0.079	4.607	4.1	< DL	12.311	0.221	0.009	< DL	9.252
28	R28	0.175	0.059	0.015	0.135	3.547	2.5	< DL	10.944	0.210	0.019	< DL	7.565
29	R29	0.122	0.086	0.009	0.551	2.341	6.9	< DL	13.702	0.000	0.003	< DL	9.481
30	R30	0.103	0.185	0.018	0.609	3.574	28.8	< DL	12.429	0.391	0.034	< DL	11.299
31	R31	0.131	0.049	0.013	1.223	2.898	16.9	< DL	13.527	0.100	< DL	< DL	7.461

No	Sample code	As	Cd	Co	Cr	Cu	Fe	Hg	Mn	Ni	Pb	Se	Zn
32	R32	0.158	0.027	0.009	0.915	1.319	18.1	< DL	8.256	< 0.001	< DL	< DL	6.464
33	R33	0.111	0.005	0.008	0.963	0.118	8.1	< DL	10.843	< 0.001	< DL	< DL	6.791
34	R34	0.114	0.019	0.015	0.903	1.317	2.8	< DL	11.362	0.103	0.104	< DL	7.355
35	R35	0.131	0.007	0.007	0.799	0.192	2.6	< DL	9.456	0.010	< DL	< DL	7.822
	**Mean**	**0.106**	**0.063**	**0.008**	**0.360**	**2.406**	**11.914**	**< DL**	**10.618**	**0.179**	**0.038**	**< DL**	**8.163**
	**Max**	**0.328**	**0.222**	**0.018**	**1.223**	**5.405**	**35.7**	**< DL**	**14.012**	**0.51**	**0.144**	**< DL**	**11.299**
	**Min**	**0.052**	**0.005**	**< DL**	**0.004**	**0.118**	**0.2**	**< DL**	**7.444**	**0.00**	**< DL**	**< DL**	**6.011**
	**SD**	**0.049**	**0.060**	**0.006**	**0.368**	**1.243**	**8.972**	**< DL**	**1.762**	**0.132**	**0.031**	**< DL**	**1.344**
36	TN1	0.185	0.099	0.621	0.364	6.074	28.303	< DL	36.339	0.730	0.114	< DL	11.402
37	TN2	0.214	0.107	0.568	< DL	7.754	39.812	< DL	29.406	0.918	0.146	< DL	8.470
38	TN3	0.149	0.181	0.791	0.242	3.007	14.345	< DL	42.650	0.370	0.076	< DL	11.653
39	TN4	0.087	0.192	0.496	< DL	2.971	10.146	< DL	20.940	0.376	0.052	< DL	7.178
40	TN5	0.135	0.090	0.797	< DL	3.615	10.737	< DL	25.505	0.647	0.114	< DL	7.882
41	TN6	0.094	0.117	0.634	< DL	4.754	9.268	< DL	22.303	0.737	0.128	< DL	9.740
42	TN7	0.084	0.258	0.760	0.099	4.397	9.066	< DL	23.500	0.948	0.108	< DL	9.196
43	TN8	0.149	0.455	0.379	< DL	3.969	14.103	< DL	19.281	0.654	0.135	< DL	7.744
44	TN9	0.110	0.115	0.495	< DL	7.559	47.314	< DL	21.398	1.243	0.088	< DL	9.051
45	TN10	0.102	0.320	0.483	0.177	5.644	10.696	< DL	35.672	1.237	0.177	< DL	11.487
46	TN11	0.107	0.480	0.491	0.071	3.750	14.579	< DL	34.008	0.651	0.058	< DL	7.265
47	TN12	0.065	0.429	0.566	0.114	5.689	9.206	< DL	25.563	1.094	0.106	< DL	9.941
48	TN13	0.207	0.168	0.369	0.029	3.681	7.195	< DL	25.593	0.852	0.073	< DL	8.154
49	TN14	0.269	0.035	0.137	0.165	2.152	15.813	< DL	21.943	0.470	0.084	< DL	6.571
50	TN15	0.137	0.143	0.040	0.111	4.932	17.350	< DL	35.278	0.607	0.078	< DL	9.928
51	TN16	0.081	0.181	0.495	0.035	3.631	10.980	< DL	28.121	0.364	0.075	< DL	8.446
52	TN17	0.080	0.017	0.021	0.038	2.337	11.232	0.004	23.287	0.289	0.104	< DL	5.341
53	TN18	0.053	0.339	0.691	0.265	3.753	7.052	< DL	31.346	0.861	0.056	< DL	7.302
54	TN19	0.145	0.017	0.087	0.191	1.491	17.378	< DL	32.532	0.211	0.078	< DL	7.358
55	TN20	0.088	0.145	0.208	< DL	3.245	8.998	< DL	30.746	0.557	0.089	< DL	7.560
56	TN21	0.112	0.214	1.369	< DL	3.302	16.116	< DL	38.138	0.435	0.070	< DL	8.116
57	TN22	0.103	0.088	0.143	0.060	1.717	9.397	0.009	20.986	0.194	0.048	< DL	6.511
58	TN23	0.087	0.117	0.125	0.030	2.493	10.326	< DL	24.973	0.295	0.059	< DL	7.759
59	TN24	0.085	0.067	0.565	< DL	3.452	13.518	< DL	27.121	0.312	0.051	< DL	8.436
60	TN25	0.187	0.025	0.490	< DL	2.599	14.575	< DL	25.771	0.251	0.054	< DL	6.635

No	Sample code	As	Cd	Co	Cr	Cu	Fe	Hg	Mn	Ni	Pb	Se	Zn
61	TN26	0.094	0.207	0.534	0.439	6.908	14.534	< DL	38.654	0.716	0.066	< DL	6.476
62	TN27	0.169	0.073	0.562	< DL	2.553	18.618	< DL	29.492	0.208	0.078	< DL	7.336
63	TN28	0.094	0.097	0.470	0.119	2.487	13.607	< DL	29.916	0.387	0.111	< DL	6.966
64	TN29	0.178	0.078	0.731	0.650	4.078	13.405	< DL	27.637	0.506	0.059	< DL	7.518
65	TN30	0.165	0.122	0.987	0.466	3.791	16.037	0.011	28.046	0.405	0.157	< DL	7.324
66	TN31	0.099	0.095	0.615	0.038	3.604	13.287	0.004	21.708	0.361	0.051	< DL	8.273
67	TN32	0.111	0.144	0.575	0.412	3.262	10.467	0.005	28.465	0.442	0.082	< DL	7.984
68	TN33	0.119	0.158	0.577	0.397	3.756	10.544	< DL	25.333	0.557	0.139	< DL	7.692
69	TN34	0.097	0.069	0.456	0.349	3.756	9.881	< DL	25.105	0.415	0.055	< DL	8.456
70	TN35	0.080	0.083	0.585	0.576	3.263	13.626	< DL	20.377	0.474	0.065	< DL	8.523
	**Mean**	**0.123**	**0.158**	**0.512**	**0.227**	**3.869**	**14.615**	**0.007**	**27.918**	**0.565**	**0.088**	**< DL**	**8.162**
	**Max**	**0.269**	**0.48**	**1.369**	**0.65**	**7.754**	**47.314**	**0.011**	**42.65**	**1.243**	**0.177**	**< DL**	**11.653**
	**Min**	**0.053**	**0.017**	**0.021**	**0.029**	**1.491**	**7.052**	**<DL**	**19.281**	**0.194**	**0.048**	**< DL**	**5.341**
	**SD**	**0.048**	**0.119**	**0.273**	**0.187**	**1.512**	**8.304**	**0.003**	**5.871**	**0.283**	**0.034**	**< DL**	**1.442**
**Mean**		**0.115**	**0.111**	**0.279**	**0.296**	**3.138**	**13.264**	**0.007**	**19.268**	**0.384**	**0.075**	**< DL**	**8.163**
**Max**		**0.328**	**0.480**	**1.369**	**1.223**	**7.754**	**47.314**	**0.011**	**42.650**	**1.243**	**0.177**	**< DL**	**11.653**
**Min**		**0.052**	**0.005**	**<DL**	**<DL**	**0.118**	**0.200**	**<DL**	**7.444**	**<DL**	**0.003**	**< DL**	**5.341**
**SD**		**0.049**	**0.105**	**0.318**	**0.295**	**1.559**	**8.688**	**0.003**	**9.717**	**0.300**	**0.050**	**< DL**	**1.384**

< DL: below detection limit
